# Assessment of the energy recovery potential of waste Photovoltaic (PV) modules

**DOI:** 10.1038/s41598-019-41762-5

**Published:** 2019-03-27

**Authors:** Charlie Farrell, Ahmed I. Osman, Xiaolei Zhang, Adrian Murphy, Rory Doherty, Kevin Morgan, David W. Rooney, John Harrison, Rachel Coulter, Dekui Shen

**Affiliations:** 1 South West College, Cookstown, Co., Tyrone, BT80 8DN Northern Ireland UK; 2 School of Mechanical and Aerospace Engineering, Queen’s University Belfast, Belfast, BT9 5AH Northern Ireland UK; 3 School of Chemistry and Chemical Engineering, Queen’s University Belfast, Belfast, BT9 5AG Northern Ireland UK; 4 Chemistry Department, Faculty of Science - Qena, South Valley University, Qena, 83523 Egypt; 5 School of Natural and Built Environment, Civil Engineering, Queen’s University Belfast, Belfast, BT9 5AG Northern Ireland UK; 6 Department of Thermal Power Engineering, Southeast University, 2 Sipailou, Xuanwu Qu, Nanjing Shi, 210018 Jiangsu Sheng China

## Abstract

Global exponential increase in levels of Photovoltaic (PV) module waste is an increasing concern. The purpose of this study is to investigate if there is energy value in the polymers contained within first-generation crystalline silicon (c-Si) PV modules to help contribute positively to recycling rates and the circular economy. One such thermochemical conversion method that appeals to this application is pyrolysis. As c-Si PV modules are made up of glass, metal, semiconductor and polymer layers; pyrolysis has potential not to promote chemical oxidation of any of these layers to help aid delamination and subsequently, recovery. Herein, we analysed both used polymers taken from a deconstructed used PV module and virgin-grade polymers prior to manufacture to determine if any properties or thermal behaviours had changed. The calorific values of the used and virgin-grade Ethylene vinyl acetate (EVA) encapsulant were found to be high, unchanged and comparable to that of biodiesel at 39.51 and 39.87 MJ.Kg^−1^, respectively. This result signifies that there is energy value within used modules. As such, this study has assessed the pyrolysis behaviour of PV cells and has indicated the energy recovery potential within the used polymers found in c-Si PV modules.

## Introduction

As the earth’s population increases, our energy demand is greater than ever, which is expected to double during the next two decades to reach around 778 exajoules (EJ) by 2035^1^. This represents a significant challenge for all demographic regions^1^. Fossil fuels like coal, natural gas, crude oil, and its derivatives are beginning to be phased out for alternative renewable energy sources, but to date are still considered the world’s primary energy source^2^. These reserves are extremely limited and can not be replenished. Burning fossil fuels also generates large quantities of carbon dioxide and pollutants which will continue to have serious environmental and health impacts. Extensive research has been carried out over recent decades to harness the energy inherent in natural phenomena such as tides, winds and sunlight. The Solar Photovoltaic (PV) industry has long been seen as one of the most important forms of renewable energy due to its ability to produce electricity without producing any subsequent emissions or pollution whilst in operation^3^. It holds significance in the electricity sector, as its potential to meet electricity demand has been widely reported; for example, Chesser *et al*. predicts that electrical micro-renewable energy systems such as solar PV could provide 30–40% of the United Kingdom’s electricity needs by 2050^4^. Currently, research in the area of photovoltaics is focused primarily on new technologies such as third generation PV^5^, optimising efficiencies and applications of solar cells by unconventional means^6–14^.

As the price of solar PV is controlled by the price of silicon, the worldwide goal of PV manufacturers is to drive the cost per Watt peak lower, thus making solar more affordable and more widely installed. As this figure continues to fall, the cost of electricity from PV technology moves ever closer to parity with grid electricity^15^. Globally, solar power now accounts for 6.3 and 1.7% of installed capacity and electricity generation, respectively^16,17^. It is expected that these figures will increase and by 2050, solar PV will facilitate between 2.5–25% of the global energy demand^18,19^.

One aspect that has been overlooked and not widely reported on is PV waste volumes due to the limited lifespan of 25–30 years for these modules^20^. With an exponential increase in annual installations, a proportional exponential increase in future PV waste is apparent even if such waste appears with a long time lag^21^. This lifespan figure would indicate as to why PV waste has not been widely reported until recently; as panels installed in the late 1980s and early 1990s are only now beginning to reach their end-of-life stage, resulting in rapid accumulation of waste. According to Kazmerski *et al*., PV modules reach their end-of-life stage when the overall power output of the module drops below 80% of the initial quoted value at the time of manufacture^22^. From the 2016 International Renewable Energy Agency (IRENA) end-of-life-management report, it is estimated that by 2030 there will be between 1.7–8 million tonnes of PV panel waste in circulation with a drastic increase to 60–78 million tonnes by 2050^23^.

As of 2012, PV modules were added to the EU’s Waste Electrical and Electronic Equipment (WEEE) directive making it law as of 2014; that manufacturers and suppliers are responsible for their end-of-life management^24,25^. Of these modules currently on the market, it has been reported that first-generation PV modules based on the semiconductor source of crystalline silicon (c-Si) have held on average between 80–90% of the market share compared to their counterpart, second-generation thin-film technology^26,27^. Commonly, unwanted electrical goods or “e-waste” as it is referred to, ends up on landfill sites along with other municipal solid waste (MSW) or is incinerated with little gas emission control, releasing toxic and carcinogenic materials into the atmosphere^28^.

The layers that make up a c-Si PV module in order of mass are as follows: glass, an anodized aluminium frame, two layers of Ethylene vinyl acetate (EVA) both, top and bottom of the silicon solar cells that encapsulate the cells, a junction box and PV backsheet (usually made from Tedlar^®^) that is located at the rear of the module^29^. Of these types of Tedlar® based backsheets, there are two main types:

TPT - Tedlar-Polyethylene terephalate-Tedlar

TPE - Tedlar-Polyethylene Terephalate-Ethylene Vinyl Acetate

Where the first letter in the abbreviation corresponds to the outermost layer of the backsheet exposed to the environment and the last letter corresponds to the most inner layer of the backsheet attached to the encapsulant layer.

This is not exclusive, however, as in recent years there have been advancements in PV backsheet construction to attempt to replace Tedlar®, which holds the highest market share of PV backsheet materials of approximately 80%^30^. Some of the alternatives that are being used are Polyesters, e-layers of ethylene copolymers, polyamides or blends with poly(methyl methacrylate) (PMMA)^31,32^. However, Tedlar® will still represent the largest share of the disposal issue; at least initially until the market shares align with a new industry standard. This indicates that Tedlar® based backsheets will represent the biggest share of the units being decommissioned until twenty-five years after a new industry standard is potentially established. Once again, reinforcing the need to focus on this composition for energy recovery (or other valorisation) from what will be a significant waste stream.

For more information and an exploded diagram on a c-Si PV module’s construction, please refer to Fig. S1 included in the supplementary information^33^. The PV module consists of these subsequent layers laminated into a very thin structure approximately 4 mm in thickness. PV modules can drop in overall power output for several different reasons. One such example is that the encapsulant degrades (also known as yellowing) over time, this modifies transmittance of light reaching the solar cells and therefore, the power generated by the module is reduced^34^. Although not fully understood, there are many sources linking and relating degradation to chemical reactions involving UV light and moisture ingress^35–37^.

Removing the polymers that encapsulate and bind to the other layers allows access to the glass, silicon and metal layers in order to further recycle these constituents inside the module. Corcelli *et al*. reported that the EVA encapsulant and backsheet polymer accounts for 6.55 and 3.6 wt.% of a PV module, respectively, with a majority of 84 wt.% being comprised of glass (74.16 wt.%) and the aluminium frame (10.3 wt.%)^38^. In general, the process of recycling PV modules starts with the manual removal of the aluminium frame and the junction box^39^. For the delamination process, the removal of EVA is the first step^33^. EVA acts as an encapsulant and electrical pottant for the silicon solar cells, protecting the components from foreign impurities, moisture and mechanical damage^40^; but also has excellent adhesion properties to the glass and backsheet layers. The removal of the EVA layer has been recognised as one of the most challenging steps in the recycling of c-Si PV modules^41^.

Several methods that have been previously employed to remove the EVA layers are dissolution using nitric acid^42^, organic solvents^43–45^, shockwave recycling^46,47^ or thermal decomposition^48^; primarily in the form of pyrolysis due to the lack of chemical oxidation or burn damage on the glass, semiconductor and metal layers^42,49^. According to the international energy agency (IEA) 2018 report, the chemical/thermal treatment of PV modules is superior to that of mechanical methods^50^. It is proposed that the pyrolysis process could contribute positively to the overall recycling rate of these modules via means of tertiary recycling by processing the waste polymers into a potential fuel source^51–53^. The products and by-products of the direct pyrolysis process could have both environmental and economic value if they are considered to be used as an alternate fuel to help delaminate further modules or used for additional applications and hence, make the process of recycling these modules self-sustaining. Herein, we further characterise the types of polymers included in c-Si PV modules by comparing delaminated samples from photovoltaic cells of used ethylene vinyl acetate (U-EVA) with virgin-grade ethylene vinyl acetate (V-EVA) alongside, used PV backsheet (UB) with virgin-grade backsheet (VB) to assess the feasibility of recycling these for calorific value using pyrolysis. In doing so this paper aims to fully identify key PV module constituent polymers and quantify, for the first time, the energy recovery potential of the polymers before PV module construction and after in the field ageing, to inform the energy assessment of recycling strategies.

## Results and Discussion

**Figure 1:**
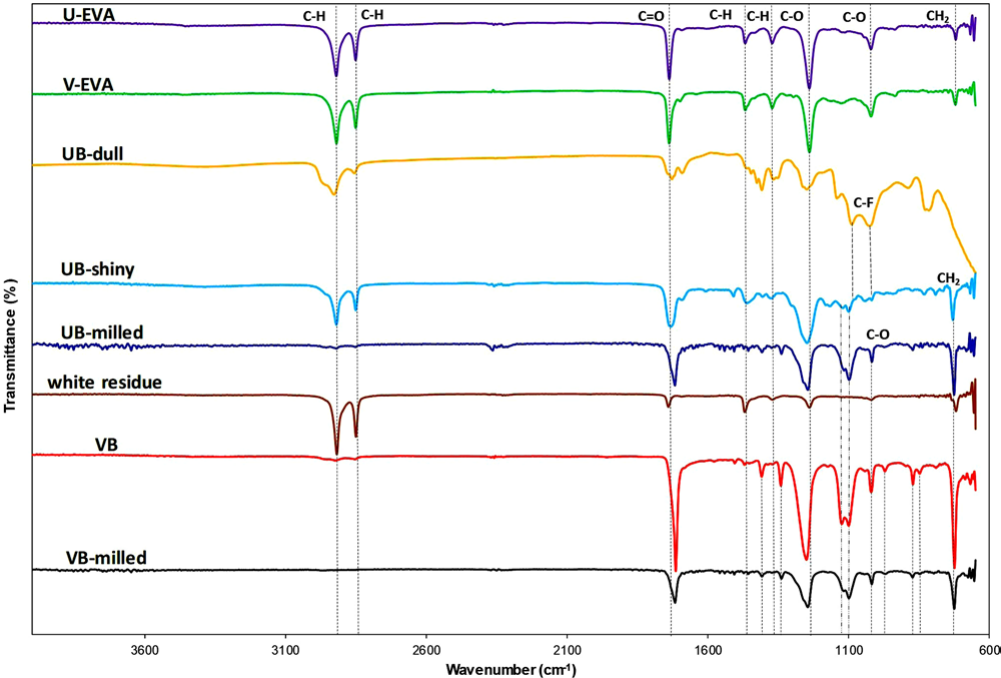
The FT-IR spectra of the EVA and backsheet samples as U-EVA, V-EVA, UB-dull, UB-shiny, UB-milled, white residue, VB and VB-milled samples.

Figure 1 contains the FT-IR spectra for all samples. Interpreting the U-EVA trace, two absorption bands are present at 2917 and 2850 cm^−1^, respectively. This is attributed to the alkane C-H bond stretching and the intensity is likely due to the long hydrocarbon chain in EVA’s overall structure^54^. The sharp band at 1739 cm^−1^ indicates the C = O stretching which is attributed to the ester group within the structure. Two bands are present at 1465 and 1368 cm^−1^, which is due to the bending of C-H bonds in a methylene group (-CH_2_) and alkane species, respectively. The most intense band in the sample is present at 1242 cm^−1^, which indicates the presence of a C-O bond in the ester functional group. Finally, two small bands occur at 1020 and 720 cm^−1^, which is attributed to C-O bond stretching and methylene (-CH_2_) rocking vibration, respectively. The bands that are present in the sample U-EVA indicate the presence of the functional groups present in EVA’s known structure and are in agreement with the work of Dias and Geretschläger^32,33^.

Interestingly, the V-EVA spectrum is very similar to that of the U-EVA sample, indicating that both the EVA layer removed from the solar cell and the virgin-grade sample (V-EVA) are similar. This is in agreement with the work of Dias *et al*., and match their findings both experimentally and the traces provided by the FT-IR database connected to the instrument for a suspected polymer match in their study^33^.

The UB-dull sample showed the most unique trace of all the FT-IR results. Interpreting the UB-dull trace, two absorption bands are present at 2922 and 2850 cm^−1^, respectively. These are attributed to the same C-H bond stretching that is present in both the EVA samples. Two further bands at 1086 and 1022 cm^−1^, respectively, are present, both of which indicate C-F bond stretching. A small band of 860–790 cm^−1^ is also present and this can either be attributed to C-X bond stretching where X represents a halogen species (thus supporting the existence of C-F bond mentioned above) or, it can also represent a C-H bond in a vinylidene species^55^. There is also a strong band present at approximately 600 cm^−1^ which is attributed to C-H bonding in an alkane species. This suggests that this particular sample of the PV backsheet could be polyvinyl fluoride (PVF) or polyvinylidene fluoride (PVDF) as these are commonly used materials for the outer layer of the backsheet that is exposed to the environment due to excellent weathering resistance and stability. Furthermore, weaker and less intense bands are present at 1732, 1686 and 1244 cm^−1^ that appear to overlap with all of the samples tested with the exception of the band at 1686 cm^−1^, as it is only present in samples UB-dull and UB-shiny. These bands are attributed to C = O bond stretching (1732 and 1686 cm^−1^) and the presence of a C-O bond in the ester group (1244 cm^−1^). This suggests that the backsheet is of Tedlar-Polyethylene Terephalate-Ethylene Vinyl Acetate (TPE) or Tedlar-Polyethylene terephalate-Tedlar (TPT) origin and the C = O & C-O functional groups contained on the next layer, the middle (PET) layer, were observed by FT-IR.

The UB-Shiny sample provides a different trace to the UB-dull sample, implying that the backsheet is a multi-layer structure as well as supporting the visual differences of either side of the PV Backsheet. The absorption bands of 2917, 2849, 1726, 1250 and 729 cm^−1^ are present again in this sample and are attributed to the C-H bond stretching (2917 and 2849 cm^−1^), C = O stretching(1726 cm^−1^), the presence of a C-O bond (1250 cm^−1^) and methylene CH_2_ rocking vibration (729 cm^−1^), respectively. The main differences between UB-shiny and UB-dull are that UB shiny does not contain the bands present at 1086 and 1022 cm^−1 ^that were attributed to C-F bond stretching. This signifies that this particular backsheet type is likely to be TPE as C-F bonds (found in Tedlar®) were identified only on one side of the backsheet by FTIR. Also, the physical appearance of both the samples is different as shown in Table S1. From the differences in the FT-IR spectra of UB-Shiny and UB-dull, it was decided that the entire multilayer structure (UB-WR) would be milled and re-tested. The UB-milled sample promoted higher intensity for bands at 1717, 1242, 1107, 1094, 1016 and 725 cm^−1^, which are attributed to C = O (1717 cm^−1^), C-O (1242 cm^−1^), C-F and methylene CH_2_ rocking vibration bonds (1016 and 725 cm^−1^), respectively. It is worth noting that bands at 2922 and 2850 cm^−1^ are not shown in UB-milled sample. This is likely due to the denaturing of C-H bonds due to the milling process as shown in Fig. 1.

The white residue sample found from delaminating the solar cell has a similar trace to V-EVA in terms of band positions at approximately 2915, 2848, 1736, 1465, 1242, 722 cm^−1^ which are attributed to C-H (2915 and 2848 cm^−1^), C = O, bending of C-H bonds, C-O and methylene CH_2_ rocking vibration bonds, respectively.

The unmilled VB sample showed a significant difference to the previous samples in that there are no bands present at 2917 and 2850 cm^−1^, respectively. This supports that the VB sample is a different material or has some different materials present in the structure compared to both EVA and used backsheet samples previously mentioned. The unmilled VB sample’s most intense band is located at 1715 cm^−1^, this corresponds to the C=O bond stretching in an aromatic ester and the intensity could be due to multiple of these functional groups. Another high-intensity band that is present in the sample spectra is found at 1247 cm^−1^. This band signifies the C-C-O bond stretching in an aromatic ester. The sample also has a double band present at 1124 and 1100 cm^−1^, respectively, which is attributed to the C-O bond in the ester group. Again, the sharp bands at 1715, 1247, 1020 and 725 cm^−1^ all overlap with the V-EVA sample. These bands, therefore, support the functional groups attributed to the aforementioned. The VB-milled sample has a direct overlap with the trace for the unmilled VB sample, concluding that the milling of the sample did not promote any additional bands in the FT-IR trace for this particular material.

In summary, similar spectra were observed for the U-EVA and V-EVA indicating that they are likely to be chemically similar. The most prevalent bonds and functional groups identified by the FT-IR experiments were C-H, C=O, C-O stretching and methylene (-CH_2_) rocking vibrations; these were observed for the majority of the samples tested. The presence of the two bands at 1086 and 1022 cm^−1^ in the UB-Dull sample would indicate that fluoropolymers are used in this particular type of backsheet (which has been delaminated from the solar cell and tested on the surface exposed to the environment) and that it is of TPE or TPT origin.

**Figure 2:**
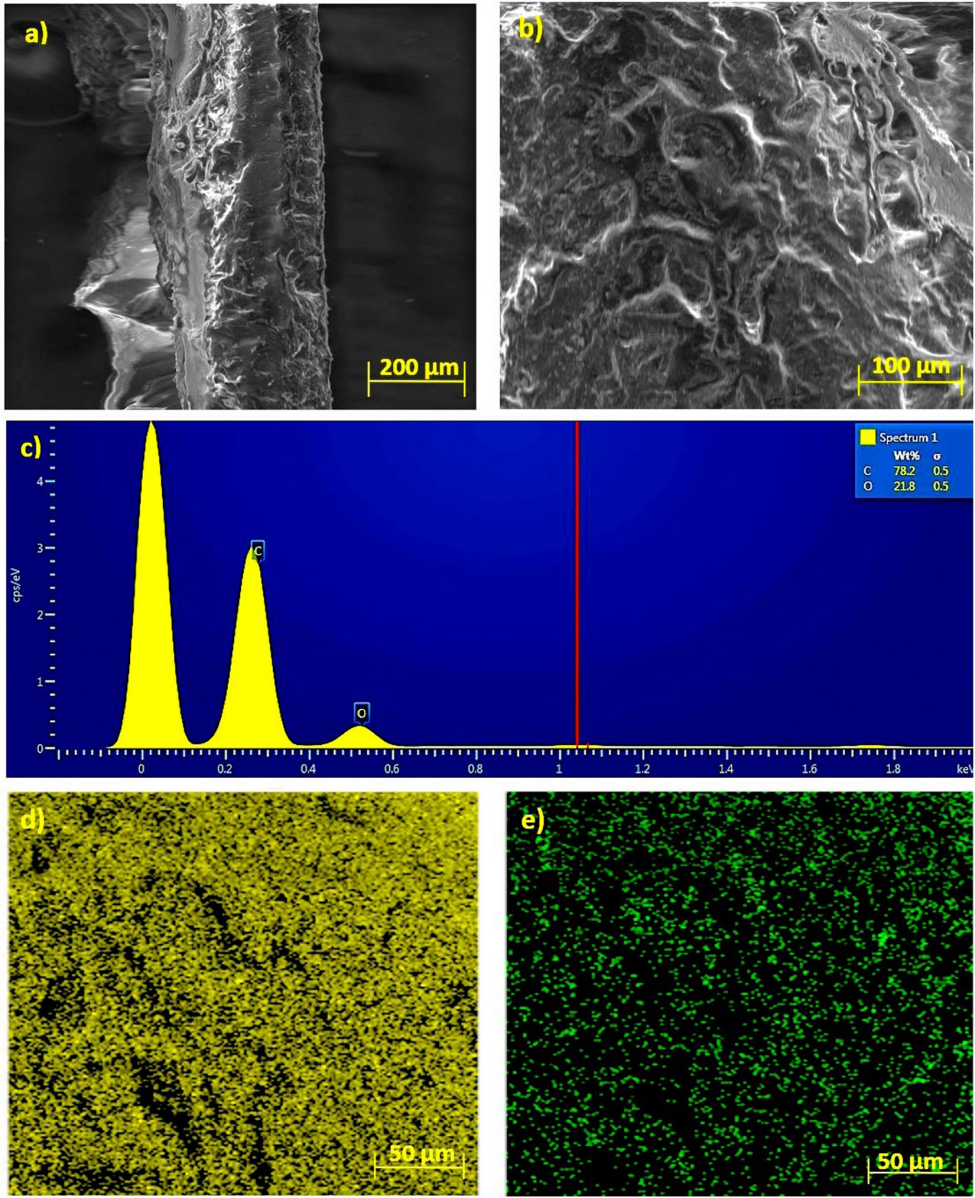
SEM images of (**a,b**) V-EVA; (**c**) EDX analysis of V-EVA; (**d**) Elemental mapping of carbon in V-EVA (**e**) Elemental mapping of oxygen in V-EVA.

Figure 2(a,b) shows SEM images of V-EVA at different magnifications while the backsheet images and relevant EDX analysis are shown in the supplementary information in Fig. S2. The results indicated existence of fluorine in samples UB-WR and UB-WOR, respectively. This is in agreement with our analysis from the FT-IR experiments on the used PV backsheet. Figure 2(a,b) both display a fascicular structure with folds and grooves on the surface of the V-EVA. This could potentially be some form of pores on the sample or due to the texture of the virgin-grade EVA prior to lamination. As the sheet of EVA has not undergone the lamination cycle there are clear textured features that can be felt on each of the surfaces. Once the EVA has gone through the lamination phase, the sheet becomes completely transparent and slightly shiny in appearance, making these textured features cease to exist as shown in the supplementary information in Table S1.

The EDX results of the V-EVA sample revealed that the surface is composed of carbon and oxygen with the percentage of 78.2 and 21.8 wt.%, respectively, as shown in Fig. 2(c). Figure 2(d,e) shows the elemental mapping of carbon and oxygen on the morphological surface of V-EVA. It is obvious that the carbon is predominant and covers most of the surface compared to the oxygen.

**Figure 3:**
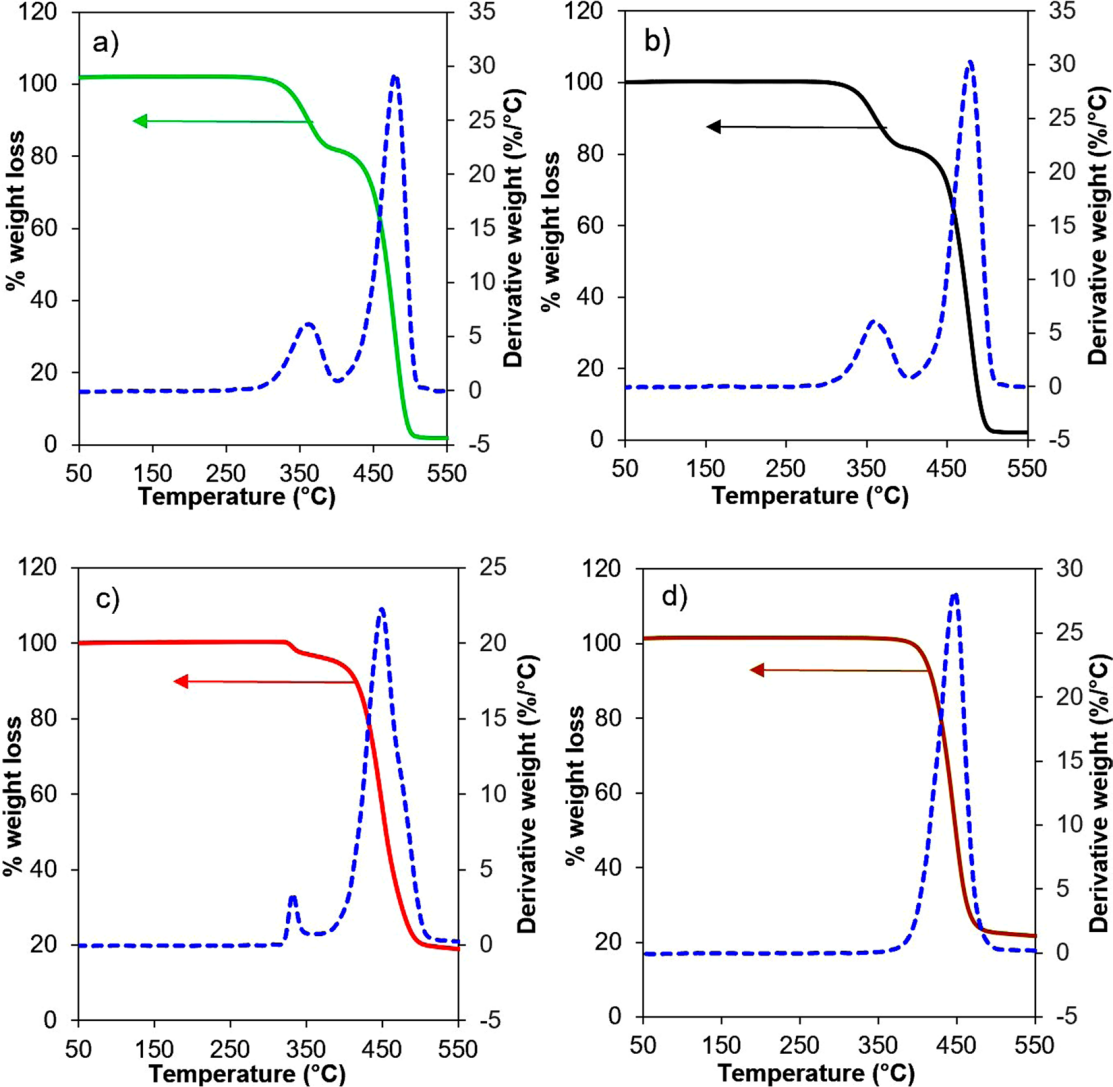
TGA/DTG curves of (**a**) U-EVA, (**b**) V-EVA, (**c**) UB and (**d**) VB over a temperature range of 50–550 °C heated at a constant heating rate of 15 °C.min^−1^ under a N_2_ atmosphere with a flow rate of 50 mL.min^−1^.

Figure 3(a–d) shows the TGA/DTG pyrolysis curves for the four samples: U-EVA, V-EVA, UB and VB under N_2_ atmosphere with a flow rate of 50 mL.min^−1^ at a constant heating rate of 15 °C.min^−1^. An air atmosphere was tested with the results shown in the supplementary information in Fig. S3.

For the U-EVA sample, a two-step decomposition was observed which is in agreement with Polanský and Rimez^56,57^ and signifies the removal of acetic acid from the vinyl acetate monomer within the EVA structure in the first decomposition stage which is known as the acetic acid shelf ^58^. This primary decomposition stage occurs at a temperature range of 310–390 °C, which corresponds to the first small peak in the DTG curve (Fig. 3(a)) with a maximum rate of weight loss of 5.98 wt% °C^−1^ at 370 °C with a weight loss of approximately 22.6 wt.%. The secondary decomposition is significantly more rapid compared to the first decomposition stage. The sample undergoes the secondary decomposition at a temperature range of 410–510 °C, with a sample weight loss of 75.7 wt.% which corresponds to the second peak in the DTG curve (Fig. 3(a)) with a maximum rate of weight loss of 29.1 wt% °C^−1^ at 475 °C.

The V-EVA exhibits a two-step decomposition and matches well with the U-EVA as shown in (Fig. 3(b)) with decomposition temperatures again at 310–390 °C and 410–510 °C for the initial and the secondary decomposition, respectively. This is in agreement with works published by Zeng^59^ but is slightly higher than results reported by Frission^60^. This is likely due to the lower heating rate that Frission used to conduct the TGA which was 5 °C.min^−1^, while herein it was 15 °C.min^−1^. It is also in agreement with the FT-IR results in Fig. 1 and confirms that the V-EVA and the U-EVA taken from the delaminated panel are similar in structure, as the decomposition temperatures are approximately the same for both of these samples. The variance was calculated against both EVA samples, using the absolute and maximum functions in Microsoft excel, showing a maximum variance of 1.28 wt.%. Again from the DTG curves shown in Fig. 3(b), there are two peaks with their maximum values corresponding to temperatures 370 and 480 °C, respectively. This DTG trend agrees with the work of Serrano *et al*.^61^. However, the results found in this study were slightly higher (370 and 480 °C as opposed to 340 and 458 °C which was reported in the literature). This is likely due to the difference in the heating rate used to conduct the experiments, as Serrano *et al*. used a lower heating rate of 5 °C.min^−1^, the decomposition temperatures and DTG peaks would be lower than what was conducted at the chosen heating rate of 15 °C.min^−1^ used herein.

Sample UB has different behaviour to the EVA samples by showing two vastly different degradation steps as shown in Fig. 3(c). Prior to the main degradation stage, it shows a minor rapid change in weight loss of approximately 3 wt.% at a temperature range of 330–340 °C. This is likely due to the degradation in the polymer as the FT-IR results in Fig. 1 suggest that UB and VB are two different materials but contain some similar functional groups. The next decomposition stage occurs at a temperature range of 390–510 °C. The residual solid content left over is ~20 wt.% of the initial sample weight and is in accordance with the mass balance experiments that were conducted in this study as the backsheet polymers produce more liquid and char fractions as by-products from the pyrolysis process compared with their EVA counterpart samples. The DTG curve in Fig. 3(c) shows a small peak at a temperature of 335 °C then a larger peak at 450 °C corresponding to a maximum rate of weight loss of 2.98 and 22.1 wt% °C^−1^, respectively.

The VB sample shows a single decomposition step in a similar range to that of sample UB (400–490 °C), but without the small shoulder that appears in the UB sample. This helps support that both backsheets are two different materials but may contain similar functional groups. This decomposition corresponds to a mass loss of ~77 wt.% of the initial mass.

Bomb calorimetry experiments were run in duplicate for all five samples tested (U-EVA, V-EVA, UB-WOR, UB-WR and VB) and each sample was dried in an oven at 60 °C for 2 hrs prior to testing. Again, due to the imperfect separation of some of the used backsheets from the solar cells, the UB sample was further divided into used backsheet with the additional white residue layer (UB-WR) and used backsheet without the white residue layer (UB-WOR). This dividing of the UB samples was also used in the CHNS experiments to determine whether or not the calorific values and elemental percentages of these samples would, in fact, be different.

The U-EVA sample produced a gross calorific value (GCV) of 39.51 ± 0.06 MJ.Kg^−1^. The GCV of the U-EVA is very high and comparable to biodiesel and heating oil which has a gross calorific value of 40.2 MJ.Kg^−1^ and 42.6 MJ.Kg^−1^ respectively^62^. The obtained GCV herein (39.51 MJ.Kg^−1^) is more than twice that of currently used energy crops such as *miscanthus*, *solanum melongena L*. and *phaseoulus vulgaris L*. (16.58, 16.52 and 17.02 MJ.Kg^−1^)^63,64^. As such, there is clearly some potential in the area of combustion/pyrolysis of PV polymers due to their high calorific value. If the heat or energy could be extracted from these polymers it would be more beneficial, both environmentally and economically than the option of landfill or direct incineration.

The V-EVA sample showed similar values to the U-EVA sample with a GCV of 39.87 ± 0.07 MJ.Kg^−1^. This again is in agreement with the FT-IR and TGA arguments that samples U-EVA and V-EVA are likely to be the same compound: Ethylene Vinyl Acetate (EVA).

UB-WR sample produced a GCV of 28.51 ± 0.04 MJ.Kg^−1^, which is lower by approximately 10 MJ.Kg^−1^ than that of the EVA, this helps add incentive to look into the development of a system to pyrolyse both the PV backsheet and EVA layers in tandem to harvest the energy and calorific values from both of these polymers, thus future work and research is needed in the areas of evolved gas analysis and utilization options.

The UB-WOR sample yielded a GCV of 22.21 ± 0.03 MJ.Kg^−1^, which is approximately 6 MJ.Kg^−1^ lower than the used backsheet with the additional white residue layer which is assumed to constitute and contribute the additional 6 MJ.Kg^−1^ unaccounted for in this particular experiment. Furthermore, the VB samples produced a GCV of 21.95 ± 0.02 MJ.Kg^−1^. This type of virgin-grade backsheet has lower calorific values than the used backsheet both; with and without the white residue layer. It only has a small difference of approximately 0.3 MJ.Kg^−1^ when comparing it to the UB-WOR sample but has a significant difference in regards to the UB-WR sample. Due to the GCV difference from that of UB-WR and UB-WOR, we can assume that both UB and VB are two different types of backsheets. This again is in agreement with the FT-IR and TGA measurements shown in Figs 1 and 3, respectively.

Finally, considering the bond dissociation energies (BDE) of the bonds broken in EVA during the delamination, the maximum threshold of energy needed to initiate the delamination process is 468.6 ± 12.6 KJ.mol^−1^, which is the BDE of O-H in acetic acid. The BDE of the R-O bond that breaks in EVA (as seen in Eq. 1), would be lower than that of the O-H bond due to the presence of a long carbon chain as opposed to hydrogen. This corresponds to an additional 0.0162 MJ.Kg^−1^ required to undertake the delamination process. Given that the calorific values obtained from the bomb calorimetry experiments of U-EVA and V-EVA (average GCV of 39.51 and 39.87 MJ.Kg^−1^, respectively) are not in the range of 0.0162 MJ.Kg^−1^, it is therefore clear that there is sufficient excess energy produced (as mentioned above) during the pyrolysis of EVA to complete the delamination, with enough surplus energy potential which can still be utilised for other applications/processes.

CHNS analysis was utilised to determine the wt.% composition of carbon, hydrogen, nitrogen and sulfur, whilst oxygen was calculated by means of difference. For all five samples tested there was no nitrogen or sulfur present in any of the samples.

Looking specifically at carbon and hydrogen content, the EVA based samples (U-EVA and V-EVA) had a higher percentage of both carbon and hydrogen than any of the backsheet samples (UB-WR, UB-WOR and VB), this explains the higher GCV of the EVA samples compared with the backsheet samples. The samples of U-EVA and V-EVA had a carbon percentage of 77.33 and 77.3 wt.%, respectively. The difference in these samples was 0.03% and can be considered negligible. The hydrogen content of these samples was 14.32 and 13.88 wt.%, respectively. The difference in hydrogen percentage still could be considered negligible in this sample (0.44%). Finally, the oxygen content of these samples were 8.35 and 8.82 wt.%, respectively, where the difference can also be negligible (0.47%). There could also be non-oxygen elements present. Once again, this implies that samples U-EVA and V-EVA are chemically similar. It is worth noting that the CHNS results for carbon of the V-EVA sample are similar to that of the EDX carbon results of the same sample (77.3 and 78.2 wt.%, respectively). This implies that both the surface composition and bulk composition of V-EVA are similar.

Both samples UB-WR and UB-WOR had significant differences in carbon and hydrogen content, where the former showed a carbon content of 65.86 wt. %, whereas the latter had shown only 58.53 wt.% (7 wt.% carbon difference). The hydrogen content for UB-WR and UB-WOR was 8.21 and 4.37 wt.%, respectively. This meant that UB-WOR had approximately half that of the UB-WR sample. It is likely that the white residue layer missing in the UB-WOR sample explains the increased percentage in both carbon and hydrogen content and the higher GCV in the UB-WR sample. It also signifies that the white residue has significantly less C and H content than the other layers; especially considering that it accounts for a significantly smaller portion of the total mass.

Furthermore, the VB sample showed that the carbon and hydrogen content was slightly higher than that of the UB-WOR sample but significantly less than the UB-WR sample. The carbon content of 59.67 wt.% was just over 1% higher than the carbon content of the UB-WOR sample. The hydrogen content of 4.47 wt.% was 0.1% higher than the hydrogen content of the UB-WOR sample. The increased carbon and hydrogen content of the VB sample did not directly translate to calorific value, as the calorific value of UB-WOR and UB-WR were both higher than the gross calorific value of VB, which could be due to the structural differences between the UB and VB samples. All results for CHNS, EDX and bomb calorimetry for all samples can be found in Table 1.

**Table 1:** Summary of bomb calorimetry results and ultimate elemental analysis (CHNS and EDX).

Samples		U-EVA	V-EVA	UB-WR	UB-WOR	VB
Bomb Calorimetry	(MJ.Kg^−1^)					
GCV 1		39.57	39.94	28.55	22.23	21.97
GCV 2		39.45	39.81	28.48	22.18	21.93
Average GCV		39.51	39.87	28.51	22.21	21.95
Elemental Composition (wt.% on dry basis)	% C	77.33	77.30	65.86	58.53	59.67
	% H	14.32	13.88	8.21	4.37	4.47
	% O	8.35	8.82	25.93	37.10	35.86
EDX Analysis (wt.% on dry basis)	% C	—	78.20	76.40	78.90	—
	% O	—	21.80	13.30	16.20	—
	% F	—	—	10.30	4.90	—

**Figure 4:**
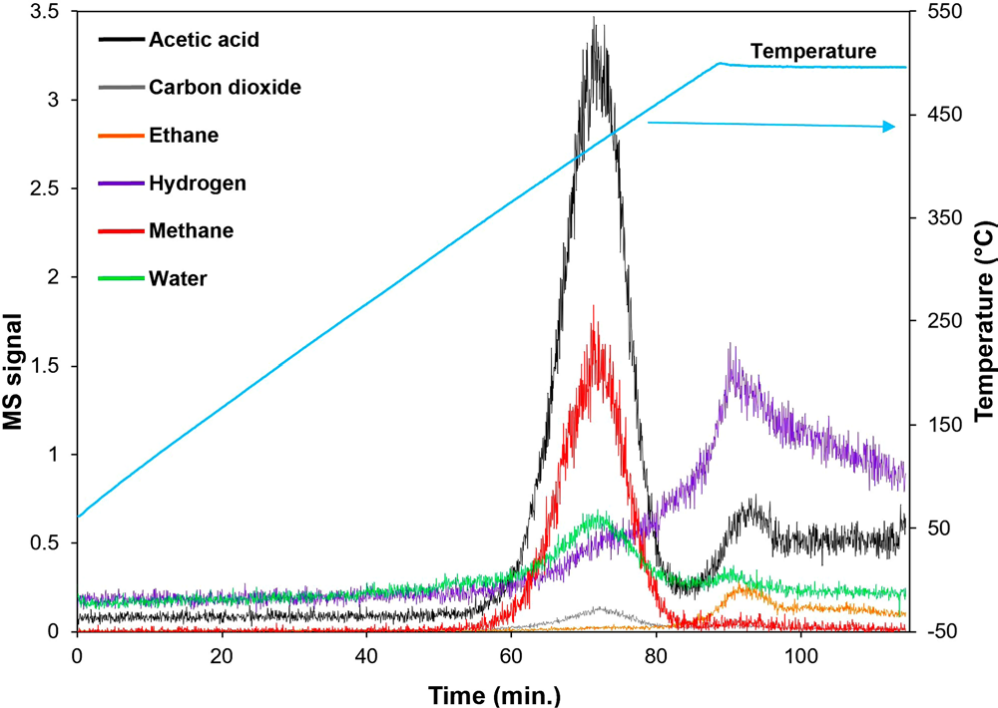
*In-situ* mass spectrometry signal of U-EVA sample heated up to 500 °C with a constant heating rate of 5 °C.min^−1^ in an inert atmosphere.

Figure 4 shows the *in-situ* mass spectrometry result where some of the main fragments contained in the gaseous phase were acetic acid, methane and hydrogen. There is a strong signal at *m/z* = 43, to which acetic acid is known to contribute, in the first decomposition stage where the first gaseous species evolves at approximately 360 °C. The results are in agreement with the TGA/DTG of U-EVA and V-EVA (Fig. 3) in which the acetic acid is released in the first decomposition stage as the acetic acid shelf ^58^. During this stage, there is also an increase in the signal of *m/z* = 13 (to which methane is known to contribute) which is likely due to the decomposition of the vinyl acetate monomer within the structure of EVA. The secondary decomposition stage occurs at approximately 470 °C, which again supports the TGA/DTG result. In this stage, there is mainly the production of hydrogen and acetic acid (*m/z* = 43 (acetic acid), *m/z* = 2 (hydrogen)). This stage signifies the breakdown of the intermediate reported by Zeng *et al*., as Poly (ethylene co-poly acetylene)^59^ into smaller species (including hydrogen and methane), as shown in Eq. 1.

1 $${(C{H}_{2}C{H}_{2})}_{X}{[C{H}_{2}CH(OCOC{H}_{3})]}_{Y}\to C{H}_{3}COOH+{(C{H}_{2}C{H}_{2})}_{X}{[CH=CH]}_{Y}$$

As *m/z* = 43 (to which acetic acid contributes) is also evolved in the second stage (470 °C) after the first main acetic acid peak (360 °C), this would explain why the first decomposition stage of the TGA only corresponds to 22.6 wt.% of the sample mass. This signifies that not all of the potential acetic acid is in fact lost in the first stage.

**Figure 5:**
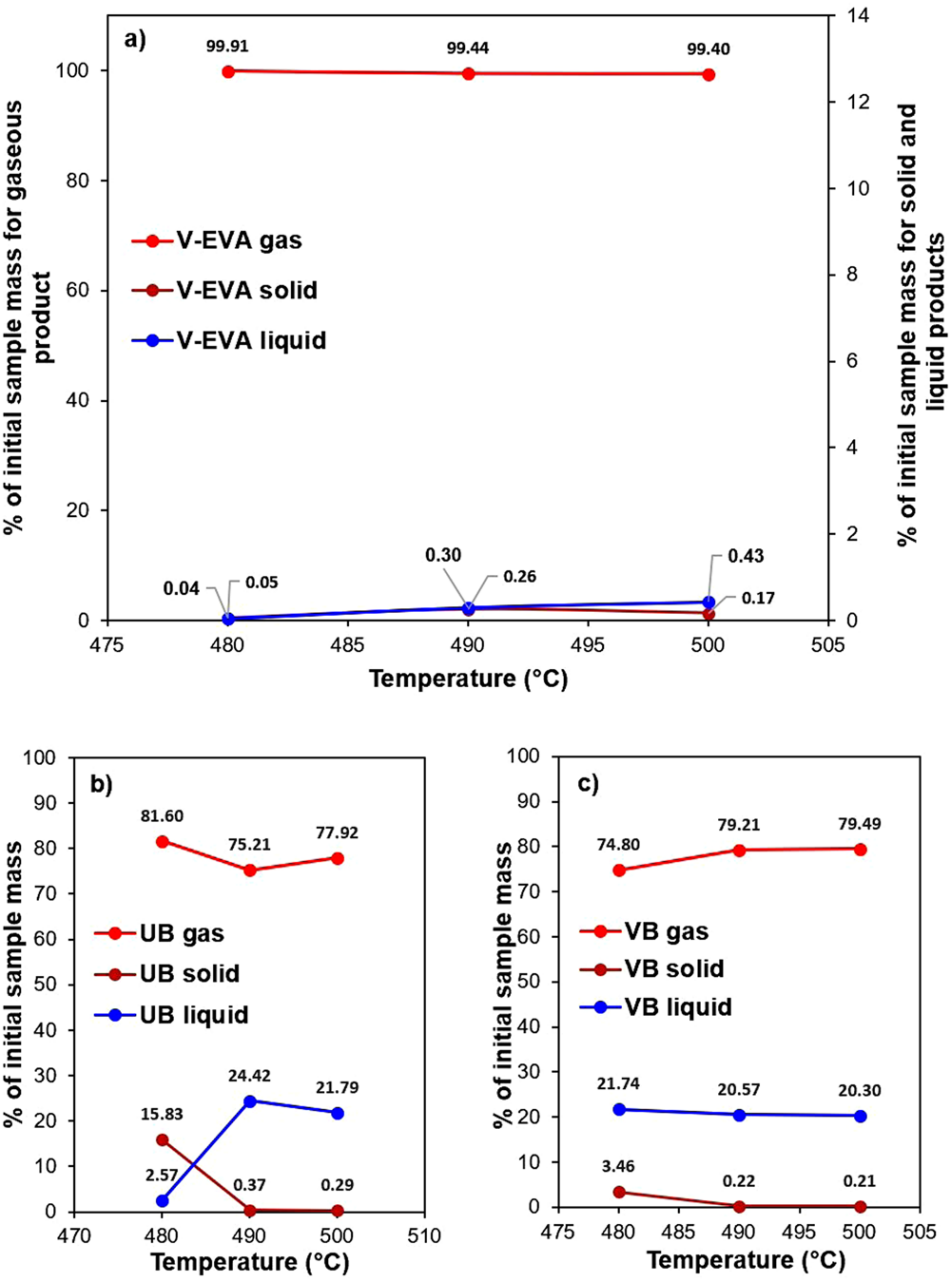
A mass balance of the gaseous, liquid and solid product distributions of: (**a**) V-EVA, (**b**) UB and (**c**) VB over a temperature range of 480–500 °C.

In order to gain an understanding of the physical pyrolysis process, mass balance experiments were carried out over the temperature range of 480–500 °C for samples V-EVA, UB and VB. This narrow temperature range was used due to a contrast of opinion on optimum pyrolysis temperature for EVA and PV backsheets published in literature^33,59,65^. These temperatures can also be used to help validate TGA/DTG and determine whether the increase in temperature benefits the product distributions. Using the conservation of mass law, the char and liquid fractions were weighed once the pyrolysis process had occurred and subtracted from the initial sample weight in order to obtain a weight for the gaseous fraction. The sample weights of all phases were converted into a percentage of the initial sample mass as shown in Fig. 5.

From the V-EVA sample, it can be inferred that the EVA polymer has the cleanest burn of all the polymers contained within the PV module. As the temperature increased from 480 to 500 °C, the gaseous fraction decreased slightly with the percentage of the initial sample mass shifting from 99.91 wt.% at 480 °C to 99.40 wt.% at 500 °C. This indicates that there was minor residual material after the pyrolysis process. This >99% gaseous fraction also supports the TGA results (Fig. 3), as the calculated value from the TGA experiment was ~1.7 wt.% residual for both EVA samples. On the other hand, the opposite trend was observed with the liquid fraction increasing from 0.03 wt.% at 480 °C to 0.42 wt.% at 500 °C.

For the UB sample, as the temperature was increased from 480 to 500 °C, the gaseous fraction decreased slightly from 81.60 to 77.92 wt.%. The relationship between the solid and liquid phases was interesting, in that when the solid content decreased from 15.83 to 0.28 wt.% when the temperature increased above 480 °C, the liquid fraction increased in a similar fashion in that the graph appears symmetrical when considering these phases. This corresponds to 24.7 wt.% total residual material left over at 500 °C, an increase from 18.4 wt.% total residual left over at 480 °C.

Finally, for sample VB the gaseous fraction increased from 74.8 to 79.50 wt.% when the temperature increased from 480 °C to 500 °C. Both the liquid and solid phases displayed similar trends to each other, in that when the temperature was increased from 480 °C to 500 °C, the percentage of initial sample mass decreased from 21.74 to 20.30 wt.% (liquid) and 3.46 to 0.21 wt.% (solid), respectively.

The grade of EVA primarily used in PV modules is Elvax 150; this corresponds to 33 wt.% vinyl acetate content. Acetic acid is removed in the first decomposition stage of EVA as shown from the TGA results, which represents 23 wt.% of the vinyl acetate monomer. This is in agreement with the TGA result which revealed that the % weight loss in the first decomposition stage (acetic acid shelf) was approximately 22.6 wt%.

The mass balance experiments showed that the EVA sample had the cleanest burn of all the polymers used in the PV module due to the >99 wt.% gaseous fraction at all three temperatures tested. This suggests that this polymer is the most feasible energy source from PV modules. Furthermore, the optimum decomposition temperature for this polymer is 480 °C. This is in agreement with Park *et al*.^65^.

## Conclusion

As time moves on exponential increases in the level of PV module waste will continue to be an increasing concern to manufacturers, consumers and recycling specialists alike. Herein, we report a comprehensive characterisation study used as a preliminary, forward-thinking step in research and development towards an energy harvesting and recycling solution to deal with an imminent increase of PV module waste in the future. An indication of the potential energy inherent from the used polymers of decommissioned or end of life PV modules is discussed. Not only can it have energy value, but it could also aid in the delamination phase with relatively clean results compared to other chemical and mechanical methods. On the basis of weight percentages of EVA and PV backsheets per PV module reported by Corcelli *et al*. (6.55 and 3.6 wt.%, respectively)^38^, the weight of an average 60 cell c-Si PV module being approximately 18.3 Kg and the estimated 60–78 million tonnes of PV module waste by 2050 forecasted by IRENA^23^; the calorific values obtained for this study further show that a potential of 155.27–201.86 and 61.58–80.06 petajoules (PJ) can be found within the used EVA and backsheet polymers, respectively. Some key findings and novelty in the work outlined are as follows:

- The calorific values of all the polymers contained in a c-Si PV module were determined using bomb calorimetry. U-EVA and V-EVA had similar high calorific values (39.51 and 39.87 MJ.Kg^−1^, respectively). This is similar to the calorific value of biodiesel and natural gas (38.7 and 39.8 MJ.Kg^−1^, respectively^62^) and helps strengthen the argument that these polymers can undergo tertiary recycling (such as pyrolysis) to create a fuel source to help delaminate further PV modules and help contribute positively to the circular economy and the overall recycling rates of these modules. It also confirms that both the aged and virgin-grade material are chemically similar.
- FT-IR results confirmed that both PV backsheets were of different composition which has provided insight into the make-up of the individual layers of the backsheets and confirmed that both EVA samples contained the same functional groups and are likely to be chemically similar.
- Evolved gas analysis from the pyrolysis of EVA helps further the understanding of the overall pyrolysis degradation mechanism of the encapsulant polymer showing the individual fractions of species in the gaseous phase and helps validate the TGA/DTG.
- To the best of our knowledge, elemental analysis (EDX and CHNS) conducted on these polymers were previously unreported. These have helped support FT-IR results, confirmed fluorinated species in the UB sample and further confirms the different backsheet compositions.
- Comprehensive mass balance tests were conducted on the pyrolysis product distributions and how these distributions are affected with temperature. This is in agreement with the TGA/DTG results and identifies how cleanly these polymers would pyrolyse while indicating how the material might behave if used as a fuel application.

## Materials and Methods

### PV cell preparation

Two used JA Solar (JAM6(L)-60–285/PR) monocrystalline silicon PV modules with broken glass were obtained for the experimental work herein. In order to get the sixty solar cells contained within the module separated for experimentation, the module was cut in square orientation using a water jet cutting machine (OMAX) in the gaps between each of the cells in order to preserve the constituents in the thin laminated structure. Each sample on average had dimensions of approximately 160 × 160 × 4 mm. For more information on the module, cutting process and subsequent cut cells please refer to Fig. S4 in the supplementary information provided. Due to the strong bonding capability of the EVA encapsulant in the solar cell, it was impossible to separate the layers cleanly at room temperature and so, a well-controlled thermal process was used. The thermogravimetric analysis (TGA) results of the decomposition of EVA and the PV backsheets confirmed that the thermal process employed did not exceed or operate near decomposition temperatures so as not to alter the overall structure of the EVA and backsheet layers^65,66^. Following extensive testing, it was determined that the solar cells were to be added to a genlab oven for 10 minutes at 190 °C for optimum results in removing the polymeric material by softening some of the polymers in the laminate structure. Once the cells were removed from the oven, a small incision was made to the side of the solar cell just above the backsheet layer. This was repeated for each side of the cell until the backsheet was able to be peeled off and removed manually. Typically this allowed for easy separation however, there was variability in the results due to the cooling time which was dependent on the time taken to delaminate the backsheet from the structure. Most samples allowed for the easy separation of the entire backsheet structure whereas others had some thin white residual material left on the surface of EVA. In order for clarification when characterising the used panels, control samples were characterised for each outcome: a perfect backsheet separation with the residue layer intact (denoted UB-WR), the white residue layer as a separate sample and the backsheet without the residue (UB-WOR).

It is worth noting that the UB-WR sample had two very distinct differences in both sides of the polymer where one side visually appeared shiny and the other dull. For more information and images on all of the samples prepared for this study, please refer to Table S1 included in the supplementary information. As the FT-IR results show the unique traces of each side of the polymer, it was decided to mill both the backsheet samples (denoted UB-milled and VB-milled) to potentially promote additional bands that were not picked up by the instrument when testing the bulk material. The milling was carried out using a Retsch SM300 cutting mill at the lowest programmable speed of 700 RPM.

### Material characterisation

Fourier Transform Infrared Spectroscopy (FT-IR) was conducted using a Jasco (FT/IR-4100typeA) spectrometer run in percentage transmittance (% T) mode with an attenuated total reflectance (ATR) attachment and a triglycine sulfate (TGS) detector with a resolution of 4 cm^−1^. Scanning Electron Microscopy (SEM) was carried out on a FEI Quanta 250 FEG MKII with a high-resolution environmental microscope (ESEM) using XT Microscope Control software and linked to an energy-dispersive x-ray (EDX) detector. The Everhart-Thornley Detector (ETD) was used in SEM analysis in order to detect the secondary electrons emitted from the sample. The EDX used was a 10 mm^2^ EDX silicon drift detector (SDD) detector-x-act from Oxford Instruments which utilises Aztec® EDX analysis software. Both systems used the same chamber.

TGA was performed using a Netzsch STA 449 C Jupiter instrument from 25 to 550 °C with a constant heating rate of 15 °C.min^−1^, in a N_2_ atmosphere with a flow rate of 50 mL.min^−1^.

Bomb calorimetry was conducted using a Parr 6200 oxygen bomb calorimeter. This instrument was used to determine the calorific values of the different polymer samples in the PV module. The bomb calorimetry experiments were run in duplicate for all samples tested and each sample was dried in an oven at 60 °C for 2 hrs prior to testing.

Elemental (C, H, N, S) Analysis was performed using a Perkin Elmer PE2400 CHNS/O Elemental Analyzer. A weighed dried sample was combusted in a tin sample crucible at 975 °C. The oxygen content was calculated by difference from the data obtained by a Perkin Elmer PE2400 CHNS/O Elemental Analyzer machine.

An *in-situ* pyrolysis experiment of a U-EVA sample was conducted where the gas evolution was monitored via mass spectrometry using a Hiden analytical HPR-20. The MS performed under a vacuum atmosphere and *in-situ* detected the characteristic fragment ion intensity of the evolved gas during the pyrolysis according to its mass to charge ratio (*m/z*) qualitatively. The selected ion recording mode was used to detect the MS signals of certain molecular ions marked accurately for the representative gas species such as *m/z* = 43 (acetic acid), *m/z* = 44 (carbon dioxide), *m/z* = 2 (hydrogen), *m/z* = 13 (methane) and *m/z* = 18 (water).

A crucible containing a U-EVA sample was fitted in a fixed bed reactor and was placed in a tube furnace with a constant heating rate of 5 °C.min^−1^ up to a final temperature of 500 °C and held for 30 minutes in an Ar atmosphere with a flow rate of 19.8 mL.min^−1^. The internal reference gas used for the mass spectrometer was a 5%Kr/Ar mixture with a flow rate of 2.98 mL.min^−1^. The fixed bed reactor temperature was monitored using a type K thermocouple and recorded using Pico Log software.

Mass balance experiments were carried out to determine the pyrolysis product distributions of the gaseous, liquid and solid products over three temperatures (480, 490 and 500 °C), respectively. All experiments were carried out in a N_2_ atmosphere with a purge rate of 20 mL.min^−1^ with a constant heating rate of 15 °C min^−1^. The hold time was 30 minutes once the reactor had reached the final temperature for each individual experiment.

## Supplementary information

ESI
