# Supplementary material for: Assessment of the energy recovery potential of waste Photovoltaic (PV) modules
Source: Sci Rep. 2019 Mar 27;9:5267. doi: 10.1038/s41598-019-41762-5 (PMC6437152; doi:10.1038/s41598-019-41762-5)
Supplement: Supplementary file 1 — ESI [file 41598_2019_41762_MOESM1_ESM.docx]

Supplementary Information (SI) for

**Assessment of the energy recovery potential of waste Photovoltaic (PV) modules**

**Charlie Farrell ^a,b*^, Ahmed I. Osman ^c,d*^, Xiaolei Zhang ^b^, Adrian Murphy ^b^, Rory Doherty ^e^, Kevin Morgan ^c^, David W. Rooney ^c^, John Harrison ^a^, Rachel Coulter ^a^, Dekui Shen ^f^**

^a^ South West College, Cookstown, Co. Tyrone, BT80 8DN, Northern Ireland, UK.

^b^ School of Mechanical and Aerospace Engineering, Queen’s University Belfast, Belfast BT9 5AH, Northern Ireland, UK.

^c^ School of Chemistry and Chemical Engineering, Queen’s University Belfast, Belfast BT9 5AG, Northern Ireland, UK.

^d^ Chemistry Department, Faculty of Science - Qena, South Valley University, Qena 83523 – Egypt.

^e^ School of Natural and Built Environment, Civil Engineering, Queen’s University Belfast, Belfast BT9 5AG, Northern Ireland, UK.

^f^ Department of Thermal Power Engineering, Southeast University, 2 Sipailou, Xuanwu Qu, Nanjing Shi, Jiangsu Sheng, China, 210018

Corresponding Authors: Ahmed Osman, Charlie Farrell

Email: [aosmanahmed01@qub.ac.uk](mailto:aosmanahmed01@qub.ac.uk), cfarrell13@qub.ac.uk

Address: School of Chemistry and Chemical Engineering, Queen's University Belfast, David Keir Building, Stranmillis Road, Belfast BT9 5AG, Northern Ireland, United Kingdom

Fax: +44 2890 97 4687

Tel.: +44 2890 97 4412
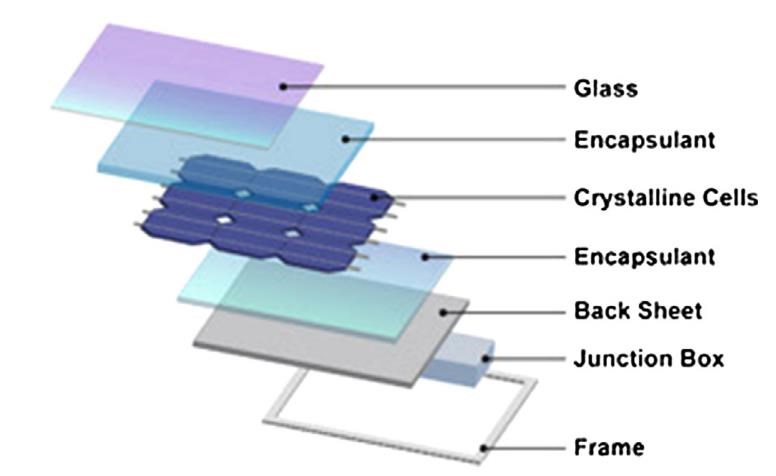


**Figure S1:** An exploded diagram of a conventional c-Si PV module and its constituent layers.^1^


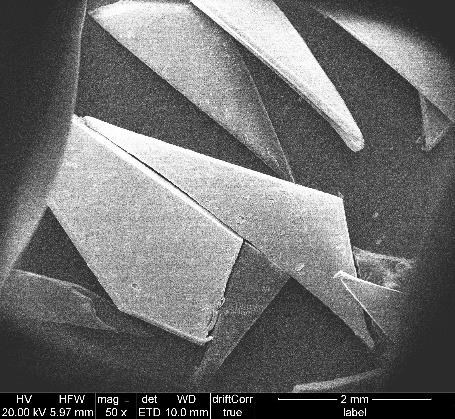

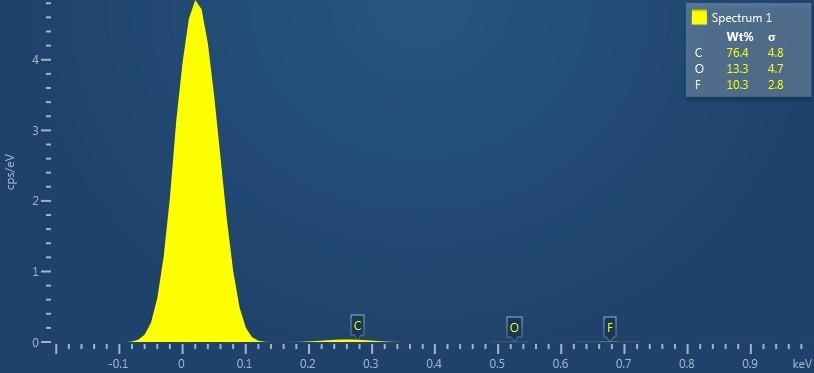

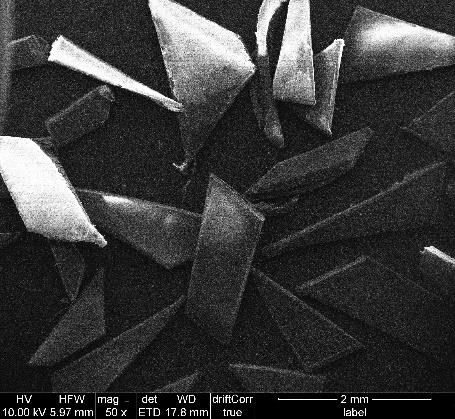

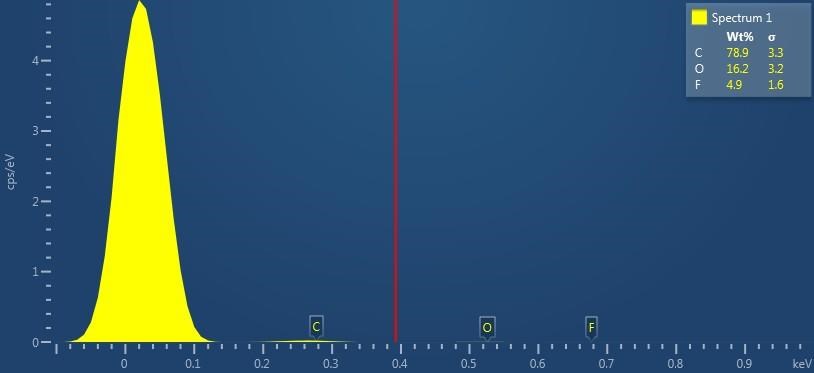


**a)**

**b**

**)**

**c**

**)**

**d**

**)**

**Figure S2:** SEM images of a) UB-WR, b) UB-WOR and EDX analysis of c) UB-WR, d) UB-WOR


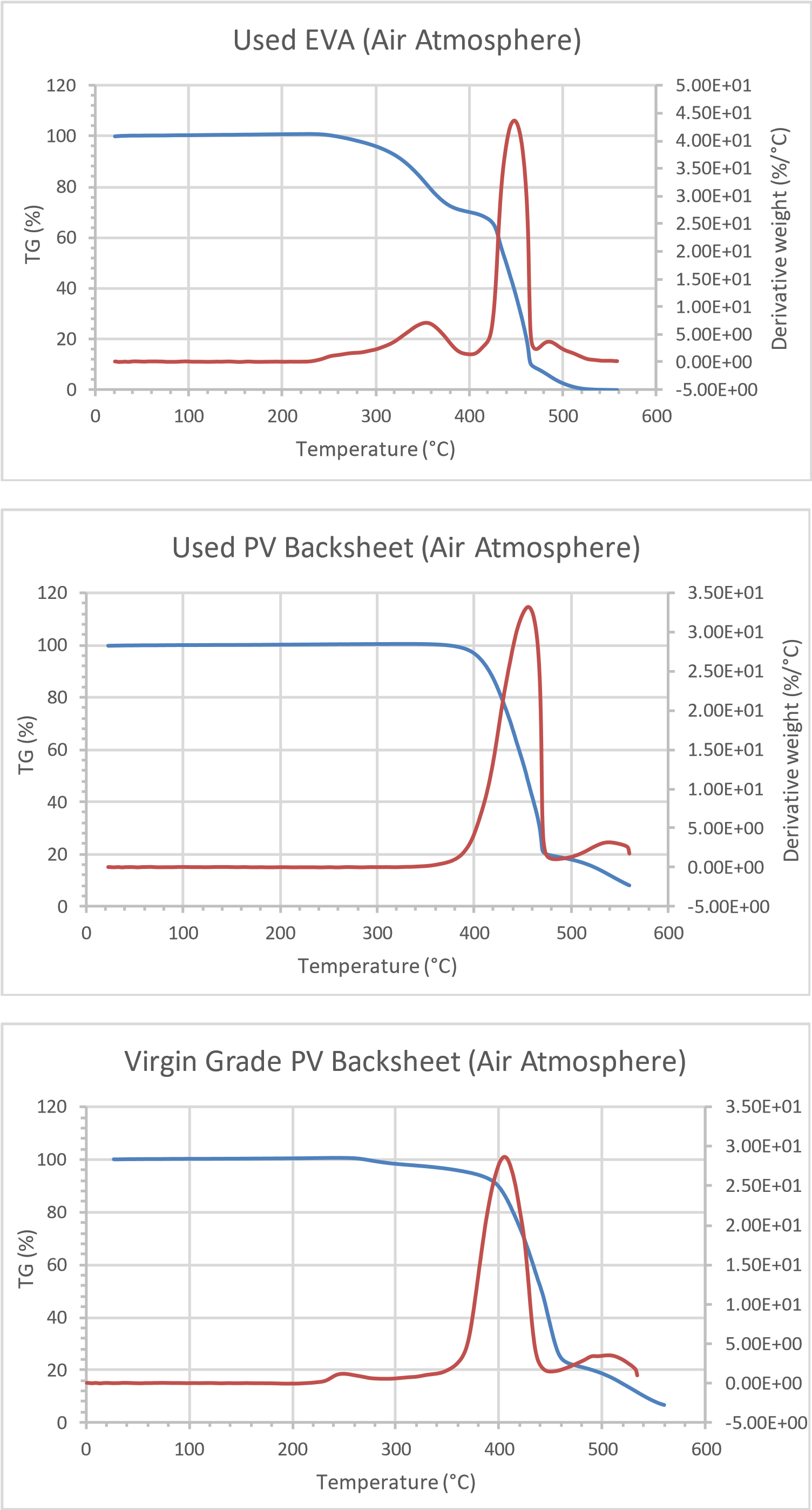


**Figure S3:** TGA/DTG curves of a) U-EVA, b) UB and c) VB in an air atmosphere.


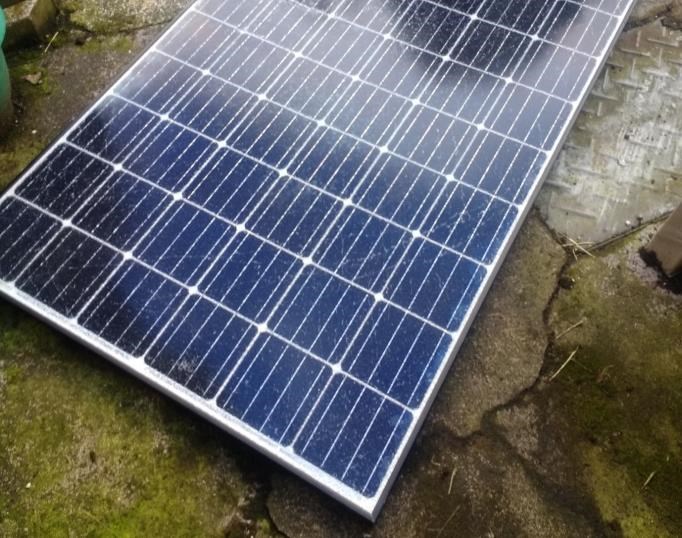

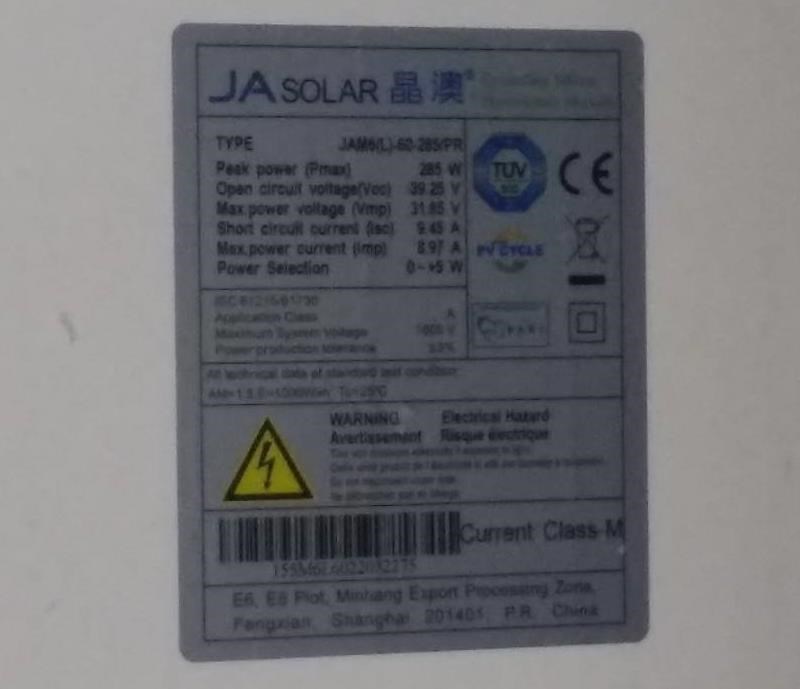


**a)**

**b**

**)**


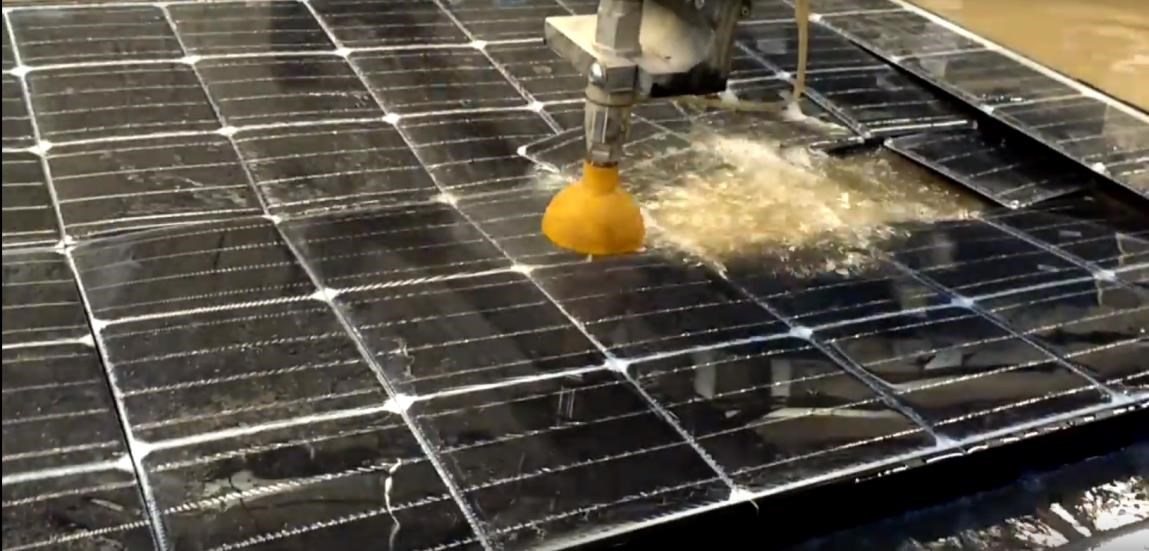


**c**

**)**

**e)**


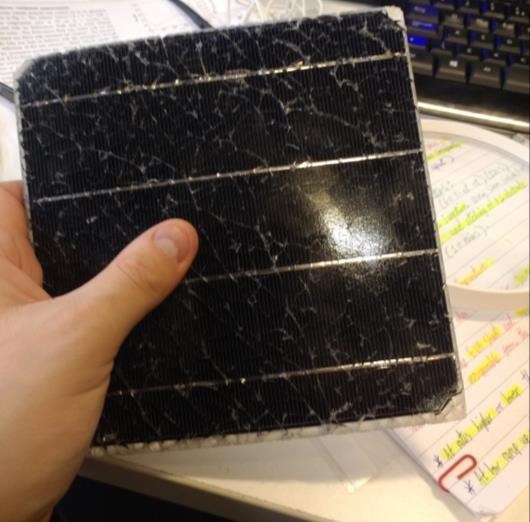

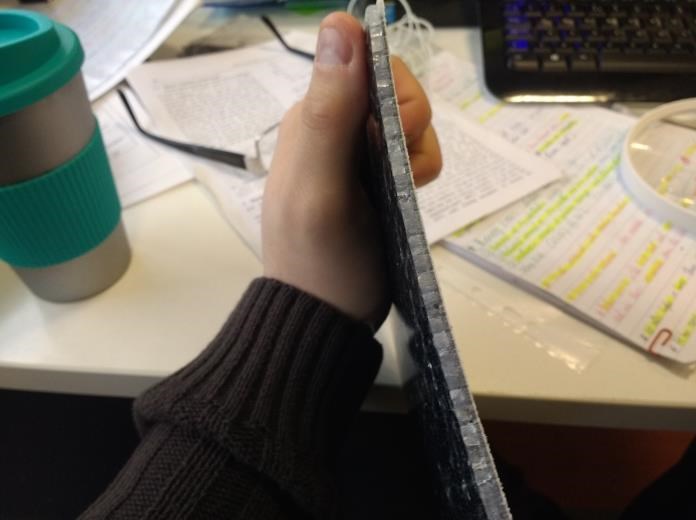


**d**

**)**

**e**

**)**

**Figure S4:** a) Monocrystalline PV module used for experiments, b) Technical information of PV module c) OMAX waterjet cutting the module into cells, images d) and e) show a face down and side profile of the cut PV cell, respectively.

| **Sample ID** | **Description** | **Image** |
| --- | --- | --- |
| U-EVA  **a)** | Used EVA taken from a delaminated solar cell. | 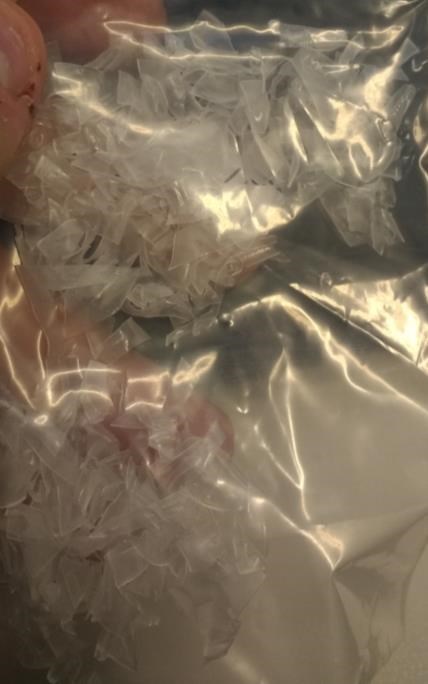  **a)** |
| V-EVA | Virgin-grade EVA. | 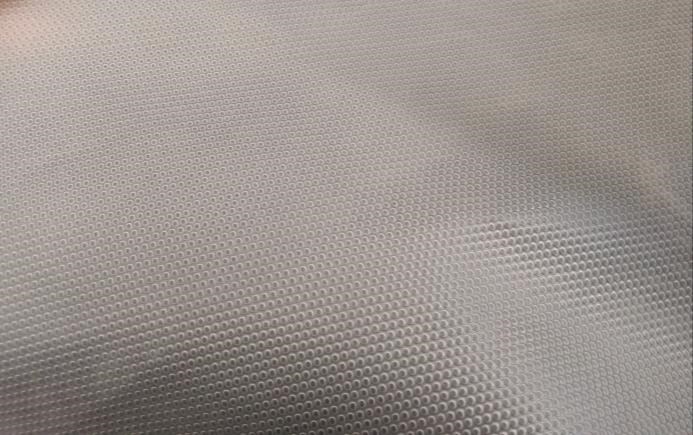 **b**  **)** |
| UB-DULL | A side of the used PV backsheet with residue intact.  (UBWR) sample that appears dull. | 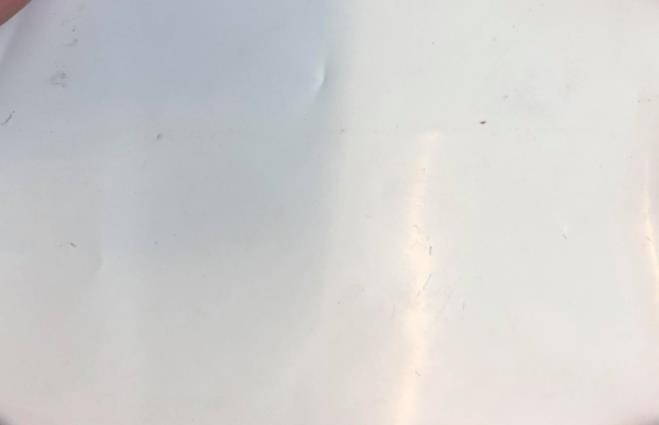 **c**  **)** |

# d)

| UB-SHINY | The reverse side of the used PV backsheet with residue intact.  (UBWR) sample that appears shiny. | 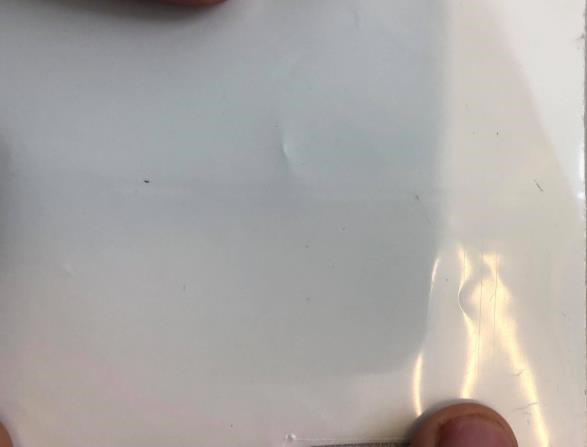  **d)** |
| --- | --- | --- |
| UB-MILLED | UBWR sample that was milled for FT-IR experiment. | 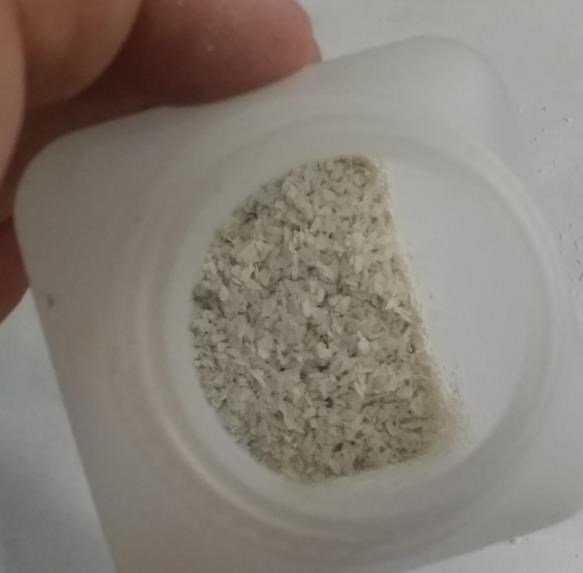 **e**  **)** |
| WHITE RESIDUE | White residue layer from imperfect separation of PV backsheet. | 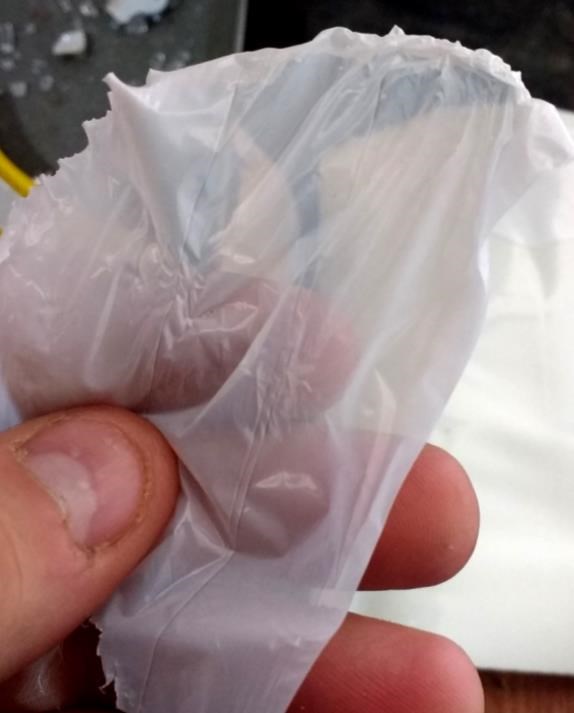 **f**  **)** |

# g)

| VB | Virgin-grade PV backsheet. | 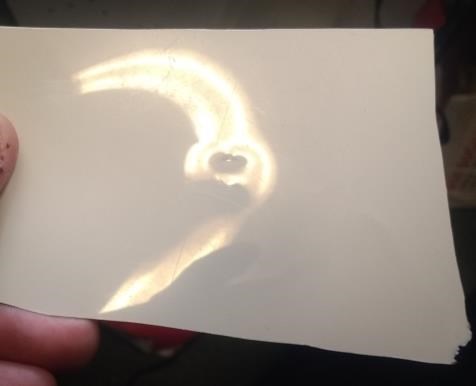  **g)** |
| --- | --- | --- |
| VB-MILLED | Virgin-grade PV backsheet that was milled for FT-IR experiment. | 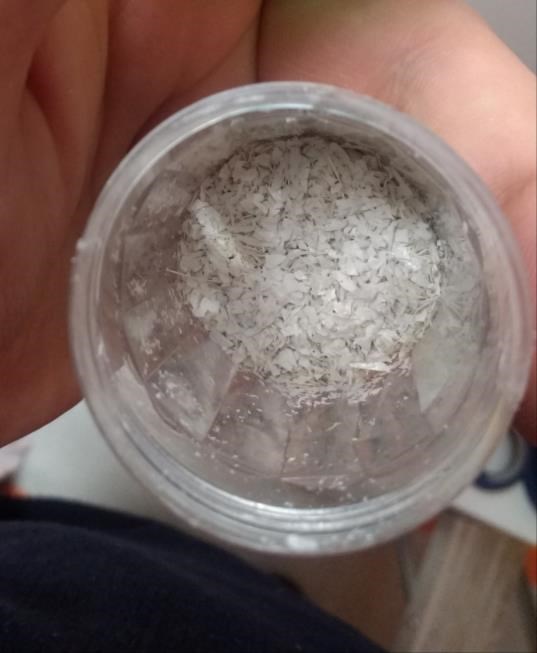 **h**  **)** |

**Table S1:** Showing sample ID, description and images for all samples used to conduct experiments.

Figure S1 Copyright details:

"Reprinted from Dias, P., Javimczik, S., Benevit, M. & Veit, H. Recycling WEEE: Polymer characterization and pyrolysis study for waste of crystalline silicon photovoltaic modules. *Waste Manag.* **60,** 716–722 (2017). doi: 10.1016/j.wasman.2016.08.036."

License Number: 4483870038266

License date: Dec 07, 2018

## References

1. Dias, P., Javimczik, S., Benevit, M. & Veit, H. Recycling WEEE: Polymer characterization and pyrolysis study for waste of crystalline silicon photovoltaic modules. *Waste Manag.* **60,** 716–722 (2017). doi: 10.1016/j.wasman.2016.08.036
